# Green/Roasted Coffee May Reduce Cardiovascular Risk in Hypercholesterolemic Subjects by Decreasing Body Weight, Abdominal Adiposity and Blood Pressure

**DOI:** 10.3390/foods9091191

**Published:** 2020-08-28

**Authors:** Beatriz Sarriá, José Luis Sierra-Cinos, Luis García-Diz, Sara Martínez-López, Raquel Mateos, Laura Bravo-Clemente

**Affiliations:** 1 Department of Metabolism and Nutrition, Institute of Food Science, Technology and Nutrition (ICTAN-CSIC), Spanish National Research Council (CSIC), José Antonio Nováis 10, 28040 Madrid, Spain; sara.martinez@universidadeuropea.es (S.M.-L.); raquel.mateos@ictan.csic.es (R.M.); lbravo@ictan.csic.es (L.B.-C.); 2 Department of Nutrition and Food Science I, School of Pharmacy, Complutense University of Madrid (UCM), Ciudad Universitaria, s/n 28040 Madrid, Spain; jlscinos@farm.ucm.es (J.L.S.-C.); diz@farm.ucm.es (L.G.-D.)

**Keywords:** green coffee, hypercholesterolemia, cardiovascular risk, weight, waist, overweight, obesity

## Abstract

In previous studies, after regularly consuming a green/roasted coffee blend, body weight, body fat%, glucose, plasminogen activator inhibitor-1 (PAI-1), resistin, leptin, ghrelin, diastolic (DBP) and systolic blood pressure (SBP) significantly changed in healthy and hypercholesterolemic subjects. However, glucagon, total-cholesterol (T-C), triglycerides (TG), LDL-cholesterol (LDL-C) and Homeostasis Model Assessment index to estimate insulin resistance (HOMA-IR) only changed in the hypercholesterolemics. This work looks into the antiobesity effects of coffee blend and into the relationship of antiobesity with the aforementioned cardiometabolic modifications in hypercholesterolemics. (1) Methods: Tricipital and subscapular skinfolds, hip, thigh, arm and waist circumference (WC) were measured in normocholesterolemic and hypercholesterolemics. To understand the relationship between cardiometabolic and antiobesity results in hypercholesterolemics, factor analysis was carried out using baseline values of the variables that changed. (2) Results: WC, WC/hip and WC/height showed significant coffee×group interaction, and in hypercholesterolemics tended to decrease. After factor analysis, three factors emerged, accounting for 29.46, 13.13 and 11.79% of variance. Only factor 1 (main loadings: WC, DBP and SBP, body weight, WC/hip and WC/height ratios, TG and ghrelin, inversely) decreased after coffee intake. (3) Conclusion: Regularly consuming green/roasted coffee may help to control body weight, and in hypercholesterolemics, may reduce cardiovascular risk by reducing abdominal adiposity and blood pressure.

## 1. Introduction

Obesity and its associated cardiometabolic alterations are currently considered an epidemic, thus, looking for treatments is of utmost importance in public health. The high cost of weight loss prescription drugs and the fear of side effects have made the use of weight loss supplements or nutraceuticals gain acceptance. Among these outstands green coffee bean extract, which constitutes unroasted coffee extracts, rich in phenolic compounds, specifically chlorogenic acids (CGA) [1,2]. However, supplement regulations based on their clinical effectiveness are not strict, they can be marketed and sold regardless of scientifically demonstrated clinical evidence, and in addition, adverse events related to dietary supplements have been reported [3]. In this context, a relevant alternative to nutraceuticals is food that is highly consumed and is naturally rich in biologically active ingredients that have shown antiobesity effects. The coffee beverage gathers both of these features, as it is the most consumed beverage worldwide after water [4] and it has shown antiobesity effects [5,6]. According to Pan et al. [7], coffee may modulate lipid accumulation in cells via cell cycle regulation during adipogenesis by inducing modifications in transcription factors and lipogenesis-related proteins in the adipose tissue of animal models, and also may decrease body weight and visceral fat in humans, thus preventing obesity. Many of these effects have been associated with coffee’s rich content in CGA, which also have antioxidant, anticarcinogenic, anti-inflammatory, and type-2 antidiabetic properties [8,9,10]. In addition to CGA, caffeine may contribute to the effects of coffee promoting weight loss due to its capacity to increase metabolic rate, energy expenditure, and lipid oxidation, as well as to its lipolytic and thermogenic activity [1]. Besides CGA and caffeine, coffee contains other bioactive compounds such as trigonelline and diterpenes (i.e., cafestol and kahweol). The amounts of all these bioactive compounds in the coffee drink depend on the coffee species, coffee roasting, extraction, and brewing procedures [11].

Roasting induces changes in the colour, taste, and aroma in coffee, related to changes in its chemical composition. During coffee roasting, many of the phenolic compounds present in the green coffee bean are lost, but in turn, compounds responsible for coffee’s organoleptic properties are formed as coffee components undergo complex Maillard reactions, producing melanoidins and other degradation products [12]. The blend of green and roasted beans is an interesting functional coffee product with more positive health effects than traditional, roasted coffee, thanks to the higher content in CGA provided by the green coffee fraction, and concurrently, it is well accepted by coffee consumers, due to the organoleptic properties provided by the roasted fraction. Several studies have been carried out in our group using a green/roasted coffee blend at the ratio 35/65 (*w*/*w*). Daily consumption of three cups of the green/roasted coffee blend (35/65), providing 445 mg of CGA/day, has shown beneficial effects against type 2 diabetes, lowering fasting glucose levels and insulin resistance, and increasing insulin sensitivity [13]. In addition, the coffee blend may prevent metabolic syndrome, reducing blood pressure, blood glucose and triglyceride (TG) levels in both healthy and moderately hypercholesterolemic [total cholesterol (T-C) 200-240 mg/dL] subjects [14]. However, a different response between healthy and hypercholesterolemic volunteers was observed for T-C, LDL-cholesterol (LDL-C), VLDL-cholesterol (VLDL-C) and TG, which decreased only in the cardiovascular risk group [15]. The lack of changes in the normocholesterolemic group is in accordance with a recent study performed in healthy subjects, after the consumption of three or five cups/day of coffee, providing 365 and 607 mg of CGA/day, respectively, for 8 weeks, as no effects on blood T-C, TG, blood pressure and some inflammatory markers were observed [16]. Similarly, in a three-arm, crossover, randomized trial, in which volunteers consumed one cup of espresso coffee/day (73 mg/serving of CGA), three cups of espresso coffee/day (219 mg of CGA), and one cup of espresso coffee plus two cocoa-based products containing coffee (8 mg of CGAs), twice per day, no significant effect on lipid metabolism (HDL-C, LDL-C and T-C, and TG) among other cardiometabolic markers was observed at any of the dosages [11]. Bearing in mind all these results, the present study is aimed at evaluating the effects of consuming the green/roasted coffee blend on anthropometric markers in healthy and hypercholesterolemic subjects, and at further understanding the relationship between antiobesity and cardiometabolic effects of the green/roasted coffee blend in hypercholesterolemics.

## 2. Materials and Methods

### 2.1. Subjects

This study was conducted following guidelines laid down in the Declaration of Helsinki for experiments in humans. It was approved by the Clinical Research Ethics Committee of Hospital Universitario Puerta de Hierro, Majadahonda in Madrid (Spain). Written informed consent was obtained from all participants and their privacy rights were always considered. Volunteers were recruited by different means: through advertisements in the Universidad Complutense de Madrid (UCM) campus; through short talks given between lectures in different schools of UCM and at the Institute of Food Science, Technology and Nutrition (ICTAN-CSIC), and through placing advertisements at health centres. The inclusion and exclusion criteria are shown in Table 1. Fifty-three people initially accepted to take part in the study, however, 52 completed it.

### 2.2. Study Design

The study was a randomized, controlled and crossover intervention, carried out in free-living people. It started with a 2-week run-in stage; afterward, volunteers were randomly assigned to begin with the coffee or control drink in the first intervention which was 8 weeks long. Subsequently, volunteers continued with a 3-week washout period, and to end, in the second intervention stage, also 8 weeks-long, they consumed the other beverage (coffee or control) they had not consumed before. During the coffee intervention, volunteers consumed a blend of green (unroasted)/roasted soluble coffee three times a day: at breakfast, between breakfast and lunch, and between lunch and dinner, always without food. Volunteers were instructed to consume the test coffee without milk or sugar. In the control intervention, instead of coffee, participants had water three times a day or an isotonic drink, free of sugar, polyphenols and methylxanthines. The green/roasted soluble coffee blend (35/65, *w*/*w*) was provided to the volunteers in unlabelled, individual sachets containing 2 g of coffee, quantity recommended by the coffee company to prepare a cup of coffee. The soluble coffee studied contained 74.2 mg/g (dry matter) of total hydroxycinnamic acids (mainly chlorogenic acid) and 20.2 mg/g (dry matter) of caffeine. The detailed composition of the coffee has been previously published [2]. The 6 g of the coffee blend daily consumed by the volunteers provided 445.2 and 121.2 mg of CGA and caffeine, respectively. As aforementioned, the green/roasted (35/65) coffee blend was studied because of its promising health effects due to the higher content in CGA provided by the green coffee and because it presents the organoleptic properties of roasted coffee. Green coffee is unroasted coffee and thus lacks such properties. This point is important as it assured the acceptability of the coffee product tested. Throughout the study, foods rich in polyphenols and methylxanthines were restricted, particularly foods rich in hydroxycinnamic acids such as chard, artichoke, eggplant, broccoli, loquats, tangerines, oranges, apricot, cherries, plums, prunes, grapes, raisins, blueberries and other fruits of the forest. Apart from foods, coffee products, mate, cocoa, and tea and derived drinks were also restricted. Moreover, whole grain products were constrained during the study, as ferulic acid and its derivatives are the most abundant hydroxycinnamic acids found in cereals, especially in the bran.

### 2.3. Dietary Control and Compliance

Study participants were indicated to keep dietary habits unchanged during the study. In order to detect any possible changes volunteers were asked to complete 72-h food intake reports in each stage of the study. Before the start of the study, volunteers were instructed on how to fill in the dietary records. Thus, in the run-in stage and the control and coffee intervention periods, volunteers were asked to complete a 72-h intake report, stating ingredients and amounts of foods consumed. When they had a scale they should register serving weights, otherwise, household measurements should be used. DIAL software for assessing diets and food calculations (for Windows, version 3.0.0.5; Department of Nutrition, School of Pharmacy (UCM) and Alceingeniería, S.A. Madrid, Spain) was applied to estimate nutrient and energy intake. Volunteers were called weekly to follow their compliance. In addition, the number of coffee sachets provided to the volunteers was counted before the start of the intervention as well as the number returned.

### 2.4. Anthropometric Measurements and Physical Activity

At the end of each stage of the study, certain anthropometric measurements (total body and trunk fat percentage) were measured using a Tanita BC-418 MA tetrapolar segmental body composition analyser, which had a weighing system included. Volunteers’ height was measured using a Holtain precision mechanical stadiometer. Body mass index was calculated [weight (kg)/height (m)^2^]. Branchial, waist, abdominal, hip and thigh circumferences and tricipital and subscapular skinfolds were measured using SECA 203 flexible tape and Harpenden skinfold caliper, respectively. Using the biometry data, body density [17] and body fat% [18] were estimated.

Apart from the dietary report, volunteers filled out a physical activity questionnaire on three representative days of each stage of the study. The questionnaire comprised the activity involved in their occupation as well as that related to sports, so they were asked to record the minutes dedicated to working, sports or other free-time activity as well as their sleeping time. Afterward, a physical activity factor (PAF) was calculated using the software ADN (for Windows, version 3.1; Department of Nutrition, School of Pharmacy, UCM) in both groups in each stage of the study. PAF corresponds to the individual’s activity level which is estimated through the physical activity questionnaire and coefficients based on the Report of FAO/WHO/UNO [19].

### 2.5. Lipid Profile. Diabetes and Appetite Biomarker Analysis

At baseline and on the last day of the control and coffee interventions, volunteers attended the Human Nutrition Unit in ICTAN and a fasting blood sample was drawn in blood tubes without anticoagulant or EDTA-coated to obtain serum and plasma samples, respectively. After separation by centrifugation the samples were frozen at −80 °C until analysis.

T-C, HDL-C, LDL-C and TG levels were determined in serum samples following reference methods or methods recommended by Sociedad Española de Bioquímica Clínica y Patología Molecular (SEQC) using a Roche Cobas Integra 400 plus analyzer (Roche Diagnostics, Mannheim, Germany). Fasting glucose concentration was analyzed using a colorimetric kit (Sprinreact).

Fasting insulin, glucagon, leptin, resistin, plasminogen-activator inhibitor-1 (PAI-1), and ghrelin were analyzed using the Bio-Rad Multiplex Diabetes kit on a Bio-Plex MAGPIX system. Analytes were measured in duplicate on a MAGPIX™ Multiplex reader fitted to a Bio-Plex Pro Wash Station. Software Bio-Plex Manager™ MP (Luminex Corporation, Austin, TX, USA) was used for data processing. Results were expressed as pg/mL plasma. Using fasting glucose and insulin data, Homeostasis Model Assessment index to estimate insulin resistance (HOMA-IR) was calculated: HOMA-IR = [Glucose (mg/dL) × Insulin (mU/L)]/405 [13].

At the end of each stage, before blood sampling, systolic (SBP) and diastolic (DBP) blood pressure was measured using an automatic arm sphygmomanometer (Pic Indolor Diagnostic, BS 150, Artsana, Italy) in triplicate, waiting for 3 min between measurements. Readings were compared and accepted if in agreement within 10–15 mmHg.

### 2.6. Statistical Analysis

Data represent mean ± standard error of the mean, unless specified otherwise. As indicated in [14,15] for sample size calculations, TC was taken as the main variable, within-patient standard deviation was assumed to be 10 and the difference between treatments 6. In addition, the statistical power was established at 80% and the significance level at 0.05. Taking all these conditions into account and considering that the study was a randomized, controlled and cross-over intervention, the sample size of 23 subjects per group was determined. Prior to statistical analysis of the results, normality of distribution and homogeneity of variance were verified using the Kolmogorov–Smirnov and Levene tests, respectively. Afterward, the general linear model (GLM) of the variance for repeated measures was used to assess the effects of consuming the coffee blend on the variables studied. The group (normocholesterolemic versus hypercholesterolemic) was taken as an intraindividual factor. Differences within each group were further studied using paired *t*-tests with Bonferroni corrections (Bonferroni Post-Hoc Test). The significance level was set at *p* < 0.05.

In order to further understand the potential relationship between the antiobesity and cardiometabolic effects of the coffee product in the hypercholesterolemic subjects, the baseline values of the variables that showed statistically significant changes as described in Sarriá et al. [14] and Martínez-López et al. [14] were included in factor analysis, except BMI as it is proportional to body weight, which was included. According to general linear model analysis carried out in the aforementioned articles, consuming coffee induced significant changes in body weight, body fat percentage, glucose, plasminogen activator inhibitor-1 (PAI-1), resistin, leptin, ghrelin, DBP and SBP. Both the coffee effect and coffee x group interaction were significant in glucagon, T-C, TG, LDL-C and HOMA-IR. In addition, in accordance with results reported in the present work, there was a significant coffee x group interaction in waist circumference, waist/hip and waist/height ratios and therefore these variables were also included in the factor analysis. To end, insulin, HDL-C, and energy and lipid intake [20] were also taken into account due to the close relationship with the aforementioned variables and their relevance in obesity.

Factor analysis was carried out to determine whether the indicated biomarkers could be replaced by a smaller number of factors to understand how they contributed to explaining the general effects of the coffee intervention. Kaiser–Meyer–Olkin (KMO) and Barlett’s test were carried out prior to the factor analysis. The factors obtained by the method of principal components analysis (PCA) were optimized using Varimax with Kaiser’s normalization rotation. Taking into consideration the percentage of total variance explained, the number of factors required to cover more than half of the variance were selected. The variables used were indexed so that values from 1 to 5 were assigned to each of the quintiles in which the total range of each variable was separated. From the coefficients calculated in the factor analysis and indices calculated for each of the variables studied in each volunteer, the value corresponding to each of the factors was calculated. This was the procedure followed so that every volunteer could be categorized by a value for each factor. The same coefficients obtained with baseline data by factor analysis, as well as cut-off points used for indexing variables, were applied to the data obtained after the coffee intervention for the same variables so that each volunteer was categorized by factors attending to baseline values and values after the intervention. To end, the Wilcoxon test was used to study if there were differences between the values of each of the factors before and after the coffee intervention.

## 3. Results

### 3.1. Volunteer Characteristics and Dietary Intake

Participant’s characteristics and dietary intake have been published in [14,15]. Briefly, volunteers were between 18–43 years old, normoweight and their blood pressure was within the healthy range (<120 and 80 mm Hg for SBP and DBP, respectively). Regarding energy and macronutrient intake, only protein intake was lower (*p* = 0.025) at the end of the coffee intervention, without any influence of the group. According to the reports, volunteers fulfilled dietary indications and food restrictions, and only one volunteer returned coffee sachets after the intervention, so compliance can be considered satisfactory.

### 3.2. Anthropometric Measurements and Physical Activity

Although body weight was similar in both groups along the study, body fat percentage, BMI and waist circumference were slightly higher in the hypercholesterolemics and thus this group presented a higher cardiovascular risk. It is noteworthy that although there were no changes in energy intake or physical activity, body weight, body fat percentage and BMI were significantly reduced after the consumption of the coffee blend without the influence of the group (Table 2). In contrast, waist circumference, waist/hip and waist/height ratios showed a significant group effect, so that only in the hypercholesterolemic volunteers these parameters were slightly reduced, although not reaching the level of statistical significance according to the Bonferroni test (Table 2). No changes were observed in the hip, thigh, arm circumferences, or in tricipital and subscapular folds.

Volunteers showed a PAF between 1.6–1.8; therefore, their physical activity was moderate [21]. The PAF observed at the end of the run-in, control and coffee stages were: 1.69 ± 0.04, 1.73 ± 0.05 and 1.73 ± 0.04 in the normocholesterolemic group and 1.70 ± 0.04, 1.69 ± 0.06 and 1.71 ± 0.05 in the hypercholesterolemic group, respectively. No statistical differences between PAF values were observed.

### 3.3. Lipid Profile. Appetite and Diabetes Biomarkers

The effects of the coffee blend on lipid profile, diabetes and appetite-related biomarkers are published in Sarriá et al. [14] or Martínez-López et al. [15]. Ghrelin levels at baseline, control and coffee stages in the normocholesterolemics were 1110 ± 62, 896 ± 51, and 715 ± 50 pg/mL and in the hypercholesterolemics 1043 ± 58, 920 ± 38 and 878 ± 56 pg/mL, respectively. The concentration of this hormone decreased after coffee intake according to the general lineal model (*p* < 0.001) and the coffee x group interaction was not significant. According to the paired test, the differences between stages were significant and followed the order: baseline > control > coffee in both groups.

The influences on glucose concentration and HOMA-IR, among other biomarkers related to metabolic syndrome, such as leptin, PAI-1, and resistin, have been described in Sarriá et al. [14]. Regarding other glucose metabolism parameters, glucagon levels changed (*p* = 0.008) with a group effect (*p* = 0.003) so that in the normocholesterolemic group, the hormone levels (pg/mL) significantly decreased (49.66 ± 1.10, 44.52 ± 1.02, 43.71 ± 1.03 corresponding to baseline, control and coffee values, respectively), in contrast to the hypercholesterolemic group (44.57 ± 1.39, 45.00 ± 0.92, 46.08 ± 2.19). Contrarily, insulin concentration (pg/mL) did not show any changes (hypercholesterolemics 8.60 ± 0.4, 9.14 ± 0.32 and 8.01 ± 0.37, and normocholesterolemics 8.94 ± 0.45, 8.79 ± 0.36 and 8.56 ± 0.42, corresponding to baseline, control and coffee values, respectively).

### 3.4. Factor Component Analysis

**Figure 1:**
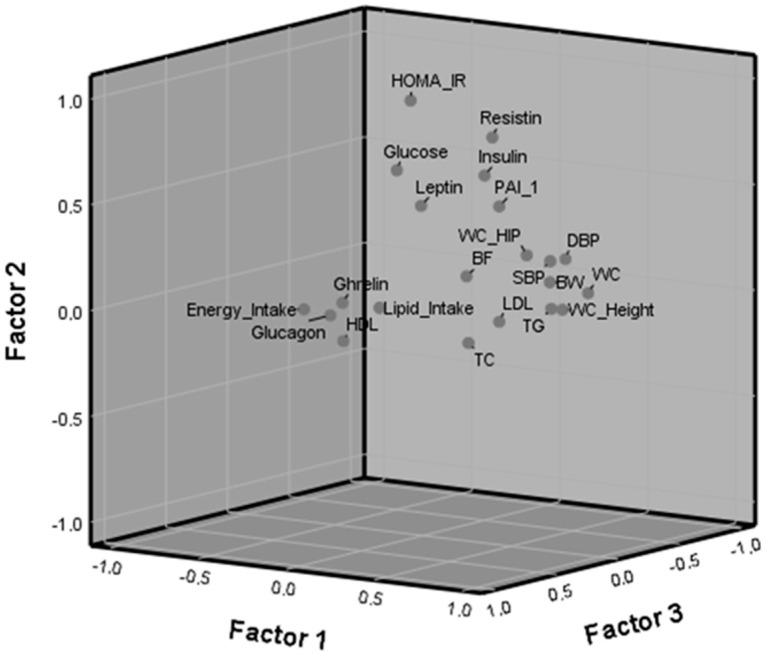
Variables in the rotated space according to the three factors (components). Higher loadings in Factor 1: waist circumference (WC), diastolic (DBP) and systolic blood pressure (SBP), body weight (BW), WC_hip and WC_height ratios and triglycerides (TG); in Factor 2: Homeostasis Model Assessment index to estimate insulin resistance (HOMA-IR), resistin, glucose, insulin, leptin and plasminogen activator inhibitor-1 (PAI-1); and in Factor 3: energy and lipid intake followed by glucagon.

**Figure 2:**
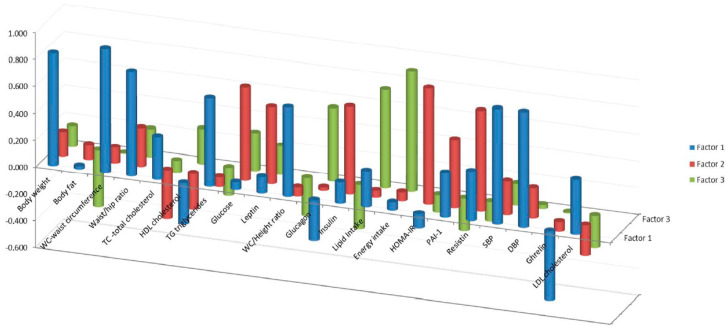
Coefficients calculated for each variable in Factor 1: waist circumference (WC), diastolic (DBP) and systolic blood pressure (SBP), body weight (BW), WC_hip and WC_height ratios and triglycerides (TG); in Factor 2: Homeostasis Model Assessment index to estimate insulin resistance (HOMA-IR), resistin, glucose, insulin, leptin and plasminogen activator inhibitor-1 (PAI-1); and in Factor 3: energy and lipid intake followed by glucagon.

Factor component analysis of the baseline values of the variables that were statistically different after coffee intake yielded three factors: factor (F1), factor 2 (F2) and factor 3 (F3) which accounted for 29.46, 13.13 and 11.79% of the variance, respectively, thus explaining 54.38% of the total variance. Within F1, the variables with higher loading were waist circumference (WC), SBP and DBP, body weight, WC/hip and WC/height ratios and TG. F2 contained main variables related to glucose metabolism, with the highest loadings corresponding to HOMA-IR, resistin, glucose, insulin, leptin and PAI-1; F3 contained variables related to dietary intake, the highest loadings were energy and lipid intake followed by glucagon (Table 3). Loadings less than 0.5 were not considered. Figure 1 shows the variables in the rotated space with the three factors (components) previously indicated and in Figure 2, the coefficients for each variable are represented separately.

The median and the interquartile range of each factor at baseline and after the coffee intervention are presented in Table 4. Only F1 decreased after the coffee intervention.

## 4. Discussion

The main findings of the present study are that the regular consumption of green/roasted coffee beverage induced reductions in body weight and fat% in both hypercholesterolemic and normocholesterolemic subjects; however, only in the hypercholesterolemics WC and WC ratios slightly decreased. Thus, this study supports that the effects of coffee are greater in individuals with higher cardiovascular risk and are modified by inter-individual variability. When the antiobesity and cardiometabolic effects of coffee in the hypercholesterolemic group were further studied through factor analysis, only the main factor that included as the variables with higher coefficients WC, DBP, SBP, body weight, WC/hip and WC/height ratios, followed by TG and ghrelin with a negative coefficient, decreased after coffee intake. Therefore, the coffee blend may reduce cardiovascular risk in hypercholesterolemic subjects mainly by inducing changes related to abdominal obesity, blood pressure and body weight.

### 4.1. Effects of the Coffee Blend on Anthropometric Measurements in Normocholesterolemic and Hypercholesterolemic Subjects

It is important to note that both the normocholesterolemic and hypercholesterolemic subjects had similar body weight at baseline and after the coffee intervention, always within the normoweight range (BMI 18.5–24.9 kg/m^2^). However, the hypercholesterolemics presented higher body fat% (2.6% higher) and WC (6.4 cm higher) at baseline than normocholesterolemics. The regular consumption of a green/roasted coffee beverage induced similar reductions in body weight and body fat%, in both groups. In contrast, WC and WC/ratios tended to decrease (not reaching statistical significance) only in the hypercholesterolemics. Thus, the present study supports that hydroxycinnamic acid-rich foods have greater effectiveness in subjects with higher cardiovascular risk [22]. The regular intake of the coffee blend here studied, which provided 445 mg of CGA/day, lowered WC by 1.2 cm in the hypercolesterolemics (contrary to the normocholesterolemics, with a WC increase of 0.5 cm), a reduction that is higher to that described by Watanabe et al. [6] in overweight men and women (0.7 cm) after consuming an instant coffee containing high amounts of CGA (369 mg CGA/serving/day) for 12 weeks, versus the control coffee (35 mg CGA/serving/day). Interestingly, in Watanabe [6], the visceral fat area, measured by computed tomography scanning, total abdominal fat area and body weight also decreased significantly in the overweight men and women. Attending to a recent meta-analysis [23] on coffee and obesity based on epidemiologic studies, higher coffee intake was associated, although statistically non-significant, with reduced risk of central obesity as defined by WC in men, but not in women, showing a non-significant but positive association. It would have been interesting to look into the differences in WC considering gender in the present study, however, the number of volunteers in the present work was too low to separate men and women subgroups. In this context, acute effects of coffee on postprandial glucose and insulin have also been shown to be modified by sex and overweight/obese status [24].

When different factors related to obesity (endocrine, metabolic and nutritional factors) were evaluated to see their relative contribution to the disease in an obese population, it was shown that dietary intake, especially fat intake, was the most important factor contributing to obesity [24]. In addition, it was concluded that abdominal adiposity distribution may induce hormonal changes and that these two factors combine to favor metabolic variations in plasma lipids, so that fat distribution may condition alterations in lipid metabolism [20]. Accordingly, the changes in abdominal adiposity observed in the hypercholesterolemics after coffee consumption, as well as the changes in resistin, leptin, and PAI-1 levels [13], could be associated with the effects on blood lipids, T-C, LDL-C and TG levels, which were significantly reduced only in the hypercholesterolemics [14]. In contrast, there were no changes in the fat distribution or the aforementioned parameters in the normocholesterolemics, in agreement with previous studies in healthy normoweight volunteers [11,16,25].

The decrease in body weight observed in both study groups may be mainly attributed to the content of CGA in the coffee blend, according to a recent review by Gökcen and Sanlier [10] among others [8,9,26]. Green coffee extract may regulate adipogenesis, as well as genes and proteins associated with lipid metabolism in white adipose tissue and liver, leading to loss of body weight. The slimming role of CGA is also supported by Roshan et al. [27], who described that decaffeinated green coffee supplementation could decrease weight about twice as much as the placebo by inhibiting adipogenesis and by contributing to controlling appetite, which was evaluated through a scored questionnaire. This outcome is in agreement with the present study, which is among the first to point to coffee controlling weight through the appetite-related hormone ghrelin. Similar results were observed after treatment with a high dose of tea extract containing epigallocatechin gallate, which reduced weight and decreased ghrelin levels in women with central obesity compared with a control group [28].

All the aforementioned effects may be associated with the consumption of three cups of the green/roasted coffee blend per day, which represents a moderate, realistic coffee consumption rate. It is important to bear in mind that the intake of certain polyphenol and methylxanthine-rich foods were restricted through the study, and energy intake and physical activity were not modified during the study. Therefore, the weight and fat loss here described may be related to the green/roasted coffee blend. Two more components of the beverage might be responsible for the observed effect on body weight (reviewed in [26,29]): (i) caffeine, increasing metabolic rate, energy expenditure, lipid oxidation, and with lipolytic and thermogenic activities; (ii) soluble dietary fiber, forming viscous solutions in the gastrointestinal tract that delay transit time and hinder the digestion and absorption of food nutrients and the reabsorption of bile salts.

### 4.2. Factor Analysis

To further understand the antiobesity and cardiometabolic effects in the hypercholesterolemic group observed in the present work and in previous studies after the intake of the coffee blend [14,15], factor analysis was carried out using the baseline values of the variables that showed significant changes due to coffee intake (body weight, body fat percentage, glucose, PAI-1, resistin, leptin, ghrelin, DBP and SBP), both a significant coffee and coffee x group interaction (glucagon, T-C, TG, LDL-C and HOMA-IR) or only a significant coffee x group interaction (WC, WC/hip and WC/height). In addition to the aforementioned variables, energy and lipid intake, which are among the most important factors contributing to obesity [20], were also included in the analysis, as well as insulin and HDL-C due to the close relationship with the other parameters.

After factor analysis, three factors emerged. F1, the main factor, accounted for 25.5% of the variance and included as the variables with higher coefficients WC, DBP, SBP, body weight, WC/hip and WC/height ratios, followed by TG and ghrelin with a negative coefficient. Therefore, within F1 were modifiable risk parameters that greatly contribute to mortality and major cardiovascular disease. Some of the associations of parameters observed within F1 are well known. Numerous studies have shown that overall obesity, defined by BMI (overall obesity) or WC (central obesity) is associated with hypertension [30,31]. Hypertension has been closely associated with obesity-related parameters not only in adults but also in adolescents, so that normal weight and overweight have shown a positive association with healthy and high BP, respectively [32]. The association between body weight and levels of TG is also in accordance with previous studies in grown-ups [33]. Alterations in levels of TG have been related to markers of central obesity, measured with simple anthropometric indices (WC and WC/ratios) and with advanced computed tomography techniques [34,35]. In contrast, the relationship between body weight and ghrelin has been less studied. It has been reported that ghrelin levels decrease in humans with obesity and metabolic syndrome and increase during weight loss, suggesting that the hormone plays a role in energy adaptation [36]. In agreement, in patients with anorexia nervosa, weight gain decreases elevated plasma ghrelin concentrations [37]. The present study supports that ghrelin is inversely related not only with body weight but also with WC and WC ratios (abdominal adiposity).

With respect to F2, which accounted for 13.13% of the variance, the variables that showed higher loadings were related to glucose metabolism: HOMA-IR, resistin, glucose, insulin, leptin and PAI-1. In addition to the well-known markers of glucose metabolism: HOMA-IR, glucose and insulin, resistin, leptin and PAI-1 are also related to type 2 diabetes (T2D). In fact, elevated levels of resistin have been associated with a higher risk of T2D [38]. Similarly, leptin has also been associated with T2D; in addition to the influence of this hormone on insulin and glucagon levels, leptin might also indirectly regulate glucose metabolism by altering levels of other hormones that are related to glucose metabolism [39]. Likewise, many studies support the implication of PAI-1 in the development of T2D, a recent meta-analysis of observational studies supports the link between PAI-1 and T2D [40].

The third main factor, F3, accounted for 11.79% of the variance and contained variables related to dietary intake. The highest loadings were energy and lipid intake followed by glucagon. According to Garaulet et al. [20], diet is the factor that explains most variability in obese patients, more than 40% is related to dietary habits, and within the diet factor, fat intake followed by energy intake are the most important factors contributing to obesity [24]. Accordingly, within F3, the higher loads corresponded to energy (0.894) and lipid intake (0.731). On the other hand, the association of glucagon and intake is well known, as glucagon suppresses appetite and also modulates lipid metabolism, and by the two routes reduces body weight and adiposity. Moreover, glucagon may promote weight loss by stimulation of energy expenditure and thermogenesis [41].

When the three factors were comparatively studied, before and after the coffee intervention (Table 4), a general decrease was observed, although only the main factor, F1, was reduced significantly, in contrast to F2 and F3 that slightly decreased without reaching the level of statistical significance. Attending to the present results, it may be inferred that in hypercholesterolemic subjects, the regular consumption of the green/roasted coffee blend mainly induces changes in abdominal obesity, blood pressure and body weight, followed by triglycerides and inversely with ghrelin. The results here presented are supported by a recent study in humans that shows that CGA may significantly decrease WC and abdominal fat, as well as SBP and appetite, and also may reduce weight marginally [27].

Limitations of this study: both the healthy and cardiovascular risk study population were normoweight. The intake of the coffee was not monitored using a coffee intake biomarker. Interindividual variability, particularly gender, was not addressed. The biological pathways associated with the main bioactive components in coffee have been described as the main pathways responsible for the reduction of cardiovascular risk; however, it cannot be discarded that other biological pathways may have been involved. Strengths of the study: the participants were homogenous regarding their lifestyle characteristics, education level and social status. Volunteers’ physical activity and dietary habits were controlled through questionnaires and did not show changes along the study. In addition, they did not smoke. Thus, several characteristics that may influence the individual response were considered. The coffee blend was well-accepted by the volunteers and self-reported compliance was high. Regarding the coffee product, sources of variability such as coffee preparation and adding sugar or dairy products were controlled by instructing the volunteers to prepare the coffee beverage only with water.

## 5. Conclusions

Regularly consuming the green/roasted coffee product may help to control weight in healthy and moderately hypercholesterolemic people, but a slight decrease in WC and WC ratios was only observed in the cardiovascular risk group. Similarly, other cardiometabolic parameters, such as lipid metabolism parameters, were only reduced in the hypercholesterolemics, not in the normocholesterolemics. Factor analysis of all the parameters that changed in the hypercholesterolemic group showed that the coffee blend reduces cardiovascular risk mainly through decreasing abdominal obesity, blood pressure, body weight, and triglycerides, and by increasing ghrelin. Therefore, the use of a green/roasted coffee blend, instead of roasted coffee, could be particularly recommended to people with cardiovascular risk within a healthy diet and to the general population to help control weight.

## Figures and Tables

**Table 1:** Volunteer inclusion and exclusion criteria.

Inclusion Criteria	Exclusion Criteria
Men and women	Suffering chronic pathology other than hypercholesterolemia
18–55 y old	Smoking
BMI 20–25 kg/m^2^	Vegetarianism
	Pregnancy in women
Attending to T-C levels in blood two groups: Normocholesterolemics (T-C < 200 mg/dL) Hypercholesterolemics (T-C 200–240 mg/dL)	Having taken dietary supplements, laxatives, or antibiotics six months before the beginning of the study

Total cholesterol (T-C); Body mass index (BMI).

**Table 2:** Effects of the consumption of green/roasted coffee on anthropometric parameters.

	Normocholesterolemics (*n* = 25)			Hypercholesterolemics (*n* = 27)			*p* Values	
	Baseline	Control	Coffee	Baseline	Control	Coffee	Coffee	Group
Body weight (kg)	63.0 ± 2.2	62.8 ± 2.8	62.5 ± 2.3	63.0 ± 2.8	62.9 ± 2.8	62.0 ± 2.8	0.017	N.S.
Body fat (%)	22.8 ± 1.4	23.7 ± 1.4	21.5 ± 1.2	25.4 ± 1.3	24.4 ± 1.2	23.6 ± 1.3	0.001	N.S.
BMI (kg/m^2^)	21.5 ± 0.5 ^a^	21.5 ± 0.5 ^a^	21.3 ± 0.5 ^b^	23.8 ± 0.5 ^a^	23.6 ± 0.6 ^a,b^	23.4 ± 0.6 ^b^	0.012	N.S.
Waist circumference (cm)	70.4 ± 1.3	70.6 ± 1.4	70.9 ± 1.4	76.8 ± 2.5	76.6 ± 2.5	75.6 ±2.5	N.S.	0.007
Hip circumference (cm)	97.2 ± 1.1	96.7 ± 1.2	96.4 ± 1.3	96.4 ± 1.3	96.6 ± 1.4	96.3 ± 1.2	N.S.	N.S.
Thigh circumference (cm)	52.0 ± 0.6	52.3 ± 0.6	51.8 ± 0.7	51.3 ± 1.0	50.9 ± 0.8	50.5 ± 0.9	N.S.	N.S.
Arm circumference (cm)	27.2 ± 0.5 ^a^	27.4 ± 0.5 ^a,b^	27.7 ± 0.5 ^b^	27.2 ± 0.8	27.3 ± 0.8	27.2 ± 0.8	N.S.	N.S.
Waist/Hip ratio	0.72 ± 0.0	0.73 ± 0.0	0.73 ± 0.0	0.80 ± 0.0	0.80 ± 0.0	0.79 ± 0.0	N.S.	0.001
Waist/height ratio	0.43 ± 0.0	0.43 ± 0.0	0.43 ± 0.0	0.46 ± 0.0	0.46 ± 0.0	0.45 ± 0.0	N.S	0.002
Tricipital fold (mm)	17.1 ± 1.2	16.7 ± 1.2	15.9 ± 1.2	16.1 ± 1.1	16.3 ± 1.2	15.9 ± 1.3	N.S.	N.S.
Subscapular fold (mm)	10.8 ± 0.7	10.5 ± 0.6	10.2 ± 0.6	13.6 ± 1.3	13.1 ± 1.2	12.9 ± 1.2	N.S.	N.S.

Values represent mean ± standard error of mean. *p* values under the coffee column correspond to the effect of consuming coffee which was studied using the general linear model of the variance for repeated measures analysis. The group was taken as an inter-subject factor, *p* values under the group column correspond to the coffee × group (normocholesterolemic vs. hypercholesterolemic) interaction. Superscripts (^a,b^) correspond to significant differences according to the Bonferroni test within either the normocholesterolemic or hypercholesterolemic group. Body mass index (BMI).

**Table 3:** Factor analysis of the variables that showed significant changes as an effect of the coffee intervention (body weight, body fat percentage, glucose, plasminogen activator inhibitor-1 (PAI-1), resistin, leptin, ghrelin, diastolic and systolic blood pressure), both coffee and coffee × group interaction (glucagon, total-cholesterol, triglycerides, LDL-cholesterol and HOMA-IR) or coffee × group interaction (waist, waist/hip and waist/height).

	Loadings of Variables by Factors		
	F1	F2	F3
Waist circumference	0.919	0.119	0.005
Diastolic blood pressure	0.862	0.225	0.029
Systolic blood pressure	0.860	0.251	0.161
Body weight	0.837	0.183	0.153
Waist/hip ratio	0.776	0.289	0.209
Waist/height ratio	0.665	−0.067	−0.285
Triglycerides	0.665	−0.072	−0.211
HOMA-IR	−0.106	0.864	−0.131
Resistin	0.368	0.749	−0.144
Glucose	0.057	0.691	0.283
Insulin	0.160	0.652	−0.330
Leptin	0.123	0.571	0.212
PAI-1	0.331	0.508	−0.241
Energy intake	0.061	0.067	0.894
Lipid intake	0.268	0.052	0.731
Glucagon	−0.303	0.025	0.545
HDL-cholesterol	−0.309	−0.267	0.262
Ghrelin	−0.513	−0.072	−0.008
Total-cholesterol	0.318	−0.364	−0.089
LDL-cholesterol	0.410	−0.227	−0.239
Body fat percentage	0.024	0.112	−0.423

**Table 4:** Comparison of factors F1, F2 and F3 at baseline and after the coffee intervention.

	F1	F2	F3
Baseline	29.08 (12.24)	5.03 (4.51)	5.89 (2.97)
After the coffee intervention	18.89 (14.86)	3.16 (4.43)	4.00 (3.50)
	*p* < 0.001	N.S.	N.S.

Values represent median (interquartile range). *p* values under each column represent the effect of consuming coffee which was studied using Wilcoxon test. Within F1, the variables with higher loading were waist circumference (WC), diastolic and systolic blood pressure, body weight, WC/hip and WC/height ratios and triglycerides. F2 contained variables related to glucose metabolism, with the highest loadings corresponding to Homeostasis Model Assessment index to estimate insulin resistance (HOMA-IR), resistin, glucose, insulin, leptin and plasminogen activator inhibitor-1 (PAI-1); whereas F3 contained variables related with intake, and the highest loadings were energy and lipid intake followed by glucagon.
